# Dr. F. T. Van Woert’s Method of Making Inlays with High-Fusing Bodies

**Published:** 1900-04-15

**Authors:** 


### DR. F. T. VAN WOERT'S METHOD OF MAKING INLAYS WITH HIGH-FUSING BODIES.

I believe that the high-fusing body is stronger than the low, and that I can manipulate it as easily as I can the low-fusing. Platinum for the matrix is rolled down to 1-1000 and 1-2000 of an inch in thickness. It is prepared by annealing it in an electric furnace on a slack-lime slab, credit for which is due to Dr. Custer, which makes it almost as soft as lead. Since I can get a metal that will stand the heat which platinum will, and of which I can make a matrix as readily as I can of gold, it is reasonable to suppose that I will select the material for the inlay which has the most strength—namely, the high-fusing body. Setting aside the question of strength, the possibility of changing the color of a high-fusing body is of not much consideration, while of a low-fusing body the change is sometimes so much that the piece is utterly worthless. In making the matrix, I take a piece of modeling compound and form it in pencil shape, sharpened at one end, and with this warmed obtain an impression of the cavity. When hardened in cold water, a counter-die of this is obtained by means of a similar pencil. The matrix is now formed as accurately as possible by pressing between this die and counter-die, and afterward perfected by burnishing into this cavity in the tooth. The procedure following that is identical with all the rest, except that I do not use any alcohol and but very little water with the porcelain body, and the water has all the gum tragacanth that it will take up cold. This is just enough to make a sticky mass of the body, and it can be manipulated on a spatula like a piece of putty. Investing it as Jenkins does, it can be cleaned free from the margins, and the resulting inlay thus made from body packed closely into the matrix is more dense than one made from a wet mass merely settled down by tapping. This is my experience. After the first baking, I remove it from the investment, put it into the cavity, reburnish the margins, and then proceed with the baking—once, twice or three times, as the case may be—and then strip the platinum from it. In the beginning I had the same difficulty as most of you have had in times past—namely, that of getting an attachment to the under side of the inlay. Cements would not hold them, because the porcelain was glazed. To grind them was almost an impossibility, as they were so small. One day I hit upon the idea of etching the under side with hydrofluoric acid, which I did by investing in beeswax the surface not to be changed. When this has been properly done, I have yet to see one that has come out. I remember a year or more ago that Dr. Ottolengui, speaking on this subject, said one of the greatest difficulties was to keep in place those thin wafer inlays. By this method they may be retained perfectly. I have no objection to the low-fusing body, other than that I get as good results with the high-fusing body, with more ease and less work.

Allow me to emphasize one point of great importance, and that is, that we must as much as possible reduce the substance that is between the inlay and the wall of the cavity. The thicker the matrix, the more cement we will have. That led me to cut down the thickness of the matrix, but by thinning rolled gold to 1-2000 of an inch it would not stand the heat even of a low-fusing body. It would burn or curl before I could be sure I had it perfectly fused. With platinum that is out of the question.

DISCUSSION.

DR. EVANS: I think Dr. Jenkins' porcelain and the subject of high and low-fusing bodies can be summed up in this way: Dr. Jenkins has introduced his porcelain more for the formation of inlays in the teeth than he has for building up the teeth or for tips, and the former is what I think will be its sphere. As to close fit of inlays to the margins of the cavity, I examined this summer the work of about all the leading operators in this branch that were at the different dental societies and elsewhere, and the work of Dr. Jenkins showed a closer adaptation to the margin of the cavity than did the high-fusing bodies. What Dr. Van Woert has succeeded in doing, I do not know; but from what he describes and the detail with which he carries out his work, it is possible that he has accomplished what the other gentlemen have not been able to do. As to the use of a high-fusing body, in one particular line of operation, I mention this. Dr. Land this summer informed me that to form a tip to an abraded tooth the best porcelain body he found for the purpose was to take an S. S. White tooth of the proper color, remove the pins, pulverize it finely in a mortar, and use this pulverized tooth as the porcelain body to form the tip. That is where the high and low-fusing bodies are going to compare. I think it will result in the lowfusing body being used for inlays, and the high-fusing for tips of teeth, etc.—*Dental Cosmos, page* 182, 1900.

